# Progress in Ophthalmology

**Published:** 1902-05-10

**Authors:** 


### Progress in Ophthalmology.

(*Continued from page* 83.)

**Extraction of Cataract in India.**[3]—The number of cataract cases operated on in India has always been a source of wonder in this country, and sometimes one has been apt to put it down to "travellers' tales," but the cataract number of the *Indian Medical Gazette* gives some figures which we suppose cannot be doubted. There is, unfortunately, never any attempt to chronicle the results as to vision.

One celebrated operator once astonished us by not being sure what was the ordinary number of the lens necessary to correct the hypermetropia left after the extraction of the cataract, although he had just finished relating how many hundred cataracts he had extracted in the last twelve months. In the *Ed. Med. Journal* they quote Smith of Jullunder, Punjab, who had 2,000 cases as less than a year's work. That during a recent month of 31 days he had operated on 688 cataracts, and on each of two particular days he had done 44 cases. "One cannot fail to be struck with the much freer use of antiseptic solutions of greater concentration than are ever used in this country." As one of the writers says, it may or may not be all very well to aim at asepsis at home, but in the vastly different conditions of Indian life and Indian patients, nothing short of antisepsis can ever be worthy of dependence. In the actual operation, in the use of atropin, or eserine, or neither, in the position of the wound, the performance or not of iridectomy, there are marked divergencies of habit and predilection as one would expect. There are differences in the recuperative powers of the patients, according to their different tribes and districts—the fact that numbers are illiterate makes it difficult to record vision with accuracy. The difficulty of so many being unable to return later probably causes unripe cataract to be attacked much more often than at home, and a form of operation to be favoured which by one means or another avoids necessity for a subsequent needling. It is no doubt this last consideration which has weighed to some extent with one surgeon of immense experience in inducing him to adopt as his routine-method, extraction in the capsule, an operation rarely performed in this country in uncomplicated cases.

**Alcoholic Amaurosis.**[4]—Frank Van Fleet, in a paper on this subject, quotes a case of a man who was addicted to the habit of periodic "sprees," after exhausting his means of supplying himself with whiskey, resorted to a solution of red pepper in water. This not satisfying him, he drank a sufficient quantity of methyl alcohol to plunge him into a profound stupor, from which he awoke blind. After a temporary improvement, he finally lapsed into a condition of permanent blindness, with total atrophy of the optic nerves. He also quotes a case of a woman who drank a large quantity of ordinary alcohol, and awoke from her stupor almost blind, but on treatment with strychnine recovered her vision in a large measure while the nerves did not atrophy. He wishes to point out that this is the only recorded case of blindness suddenly following the ingestion of ordinary or ethyl alcohol, and that as de Schweintitz suggests, methyl alcohol being cheaper is often used in the preparation of essences, as essence of anise, essence of peppermint, and essence of ginger—in this methyl or wood alcohol there seems to be something detrimental to vision. He thinks all this demonstrates to the physician two plain duties—first, to point out to the public the fact that wood alcohol is a positively dangerous substance to take internally, and which may even produce permanent blindness if the fumes are inhaled for a long time; secondly, to point out to the health authorities the fact that the cupidity and avarice of certain unscrupulous manufacturers and dealers in alcohol and alcoholic preparations may lead them to substitute methyl alcohol for the ordinary alcohol of commerce, because the former is cheaper, and that this substitution may work injury to their customers.

[3] Ed. Med. Jour., Sept., 1901. [4] Med. Rec., Jan. 18, 1902.

